# Concentrate supplementation improves cold-season environmental fitness of grazing yaks: responsive changes in the rumen microbiota and metabolome

**DOI:** 10.3389/fmicb.2023.1247251

**Published:** 2023-08-28

**Authors:** Simeng Yi, Hao Wu, Yue Liu, Dongwen Dai, Qingxiang Meng, Shatuo Chai, Shujie Liu, Zhenming Zhou

**Affiliations:** ^1^College of Animal Science and Technology, China Agricultural University, Beijing, China; ^2^College of Agriculture and Animal Husbandry, Qinghai University, Xining, China

**Keywords:** yak, rumen, microbiota, metabolomics, concentrate supplementation

## Abstract

Yak (*Bos grunniens*) is an important economic animal species on the Qinghai-Tibet Plateau. Yaks grazed in the cold season often suffer from nutritional stress, resulting in low production performance. This situation can be improved by properly feeding the grazing yaks in the cold season; however, there is still little information about the effect of different feeding levels on the intestinal microflora and metabolites of yaks. Therefore, this study aimed to explore the effect of feeding different doses of concentrate supplements on rumen bacterial communities and metabolites in grazing yaks during the cold season. Feed concentrate supplementation significantly improved the production performance and rumen fermentation status of grazing yaks during the cold season, and switched the type of ruminal fermentation from acetic acid fermentation to propionic acid fermentation. Ruminal fermentation parameters and ruminal bacterial abundance correlated strongly. At the phylum level, the abundance of *Firmicutes* increased with increasing concentrate supplementation, while the opposite was true for *Bacteroidota*. At the genus level, the abundance of *Christensenellaceae_R-7_group*, *NK4A214_group*, *Ruminococcus*, *norank_f__Eubacterium_coprostanoligenes_group*, *norank_f__norank_o__ Clostridia_UCG-014*, *Lachnospiraceae_NK3A20_group*, *Acetitomaculum*, and *Family_XIII_AD3011_group* increased with increasing concentrate supplementation, while the abundance of *Rikenellaceae_RC9_gut_ group* decreased. Dietary concentrate supplementation altered the concentration and metabolic mode of metabolites in the rumen, significantly affecting the concentration of metabolites involved in amino acid and derivative metabolism (e.g., L-aspartic acid, L-glutamate, and L-histidine), purine metabolism (e.g., guanine, guanosine, and hypoxanthine), and glycerophospholipid metabolism (e.g., phosphatidate, phosphatidylcholine, and phosphocholine), and other metabolic pathways. The strong correlation between yak rumen microorganisms and metabolites provided a more comprehensive understanding of microbial community composition and function. This study showed significant changes in the composition and abundance of bacteria and metabolites in the rumen of cool season grazing yaks fed with concentrate supplements. Changes in ruminal fermentation parameters and metabolite concentration also showed a strong correlation with ruminal bacterial communities. These findings will be helpful to formulate supplementary feeding strategies for grazing yaks in the cold season from the perspective of intestinal microorganisms.

## 1. Introduction

Yaks (*Bos grunniens*) live mainly in plateau areas above 3,000 m and are an important economic resource for plateau herders (Sapkota et al., 2022). Most yaks are raised using year-round grazing, which rarely involves hand-feeding (Long et al., 2005). The cold season on the Tibetan Plateau runs from October to May, and because of the low temperatures, the pasture does not provide enough nutrition for yak growth. This results in low productivity and a long growth cycle for yaks, with the standard age for slaughter often being over 5 years old (Long et al., 1999). However, with the improvement of economic conditions in the modern Tibetan plateau pastoral areas, yak production has also been subject to human intervention. Timely supplemental feeding of yaks during winter when forage resources are scarce is an effective means to alleviate nutritional stress in yaks (Zou et al., 2019). Bacteria in the rumen can carry out complex metabolic activities by fermenting feed, and use the resultant metabolites to reproduce, contributing to the homeostasis of the rumen environment (Clemmons et al., 2019; Xue et al., 2020). However, supplemental feeding disrupts this homeostasis because it changes the structure of the diet fed to the yak. Studies have shown that changes in diet (including the type of diet and the ratio of diet concentrate to forage) can have a dramatic effect on the ruminal bacterial community (Granja-Salcedo et al., 2016; Chen et al., 2021). Most studies on supplemental feeding of grazing yaks have focused on the effects on yak production performance. Fewer studies related to changes in the rumen microbiota and metabolome responsiveness of yaks grazing in the cool season after supplemental feeding have been published. Studies have confirmed that seasonal differences in the nutritional value of forage in pastures are an important reason for differences in the ruminal microbial communities of yaks between seasons (Ahmad et al., 2021; Huang et al., 2022). In addition, feed type and dietary concentrate ratio significantly affected the diversity and abundance of bacterial communities and the concentration of metabolites in the rumen of yaks (Liu et al., 2019; Yi et al., 2022). Therefore, it is necessary to study rumen bacteria and metabolites in cold-season supplemented grazing yaks to determine the environmental adaptation mechanisms of the yak rumen during grazing in the cold season.

Herein, we hypothesized that fermentation products, microbial communities, and metabolites in the rumen would change according to the amount of supplementation. To test this hypothesis, we characterized the yak rumen bacterial communities and metabolites using multi-omics techniques. In addition, we focused on correlations between rumen bacterial communities and metabolites, which could provide a more comprehensive knowledge base to manage cool-season grazing yaks.

## 2. Materials and methods

### 2.1. Animal feeding and management

To achieve the objective of studying the environmental adaptation mechanisms of yaks during the cold season, we chose 3 months of the cold season on the Tibetan plateau to conduct the experiment (October to December). Forty-eight 30-month-old male yaks of similar weight (204.90 ± 10.65 kg) were randomly divided into four groups of 12 yaks each. The control group was grazed only, and the remaining three groups were divided into the low supplementation group (LCS, 0.5 kg/d), the medium supplementation group (MCS, 2.5 kg/d), and the high supplementation group (HCS, 4.5 kg/d) according to the daily amount of concentrate supplementation fed to each yak. Table 1 shows the ingredient composition and chemical composition of the concentrate supplements. Supplementary Table 1 demonstrates the quality of forage during the trial period. The trial period was 105 days, divided into a pre-feeding period of 15 days and a formal feeding period of 90 days. Grazing took place at 8:00 a. m. and ended at 6:00 p. m. each day during the trial. Grass species in the pasture included Artemisia subulata Nakai, Poa crymophila Keng, and Elymus nutans Griseb. The three supplementation groups were fed half an hour before the start and half an hour after the end of grazing to ensure that each yak in the same supplementation group received the same weight of the concentrate supplement and that it was consumed in full. The yaks were allowed to drink freely throughout the experiment.

**TABLE 1:** Composition of the concentrate supplements and their chemical composition (DM basis, %).

Ingredient	Content	Chemical composition^1^	Content
Corn	44.27	DM	89.73
Wheat bran	12.06	CP	18.76
Wheat	12.88	NDF	16.03
Soybean meal	4.98	ADF	7.83
Cottonseed meal	12.28	EE	2.63
Ca(HCO_3_)_2_	8.57	Ca	0.60
NaHCO_3_	1.22	*P*	0.73
NaCl	0.87	ME (Mcal/kg)	2.58
Premix	0.87	NE_m_/(Mcal/kg)	1.74
Total	100.00	NE_g_/(Mcal/kg)	1.17

^1^DM, dry matter; CP, crude protein; NDF, neutral detergent fiber; ADF, acid detergent fiber; EE, ether extract; ME, metabolizable energy; NE_m_, net energy requirement for maintenance.

### 2.2. Sample collection and storage

All yaks were weighed before the first day of supplementation in the official period, which was recorded as the initial weight. All yaks were weighed before morning feeding on the last day of the experiment, which was recorded as the final weight. Growth performance was calculated based on final and initial body weights. One-way ANOVA model analysis of variance (ANOVA) was performed using SPSS software, and multiple comparisons were performed using Duncan’s method, with *P* < 0.05 being considered significant and 0.05 < *P* < 0.01 being highly significant.

All yaks were numbered (1 to 48). Random sampling was performed using SPSS software, and six numbers were randomly selected from each group of 12. The rumen fluid from the yaks represented by the six numbers was selected for all subsequent analyses. Select the yak rumen fluid represented by six numbers for all subsequent analyses. Six yaks in each group were randomly selected for sampling. Before morning feeding on the last day of the experiment, rumen fluid was collected transorally through a rumen cannula with an initial filter in the front port using a syringe with a volume of 50 mL. The aspirated rumen fluid was filtered through four layers of sterile gauze and its pH determined. The filtered rumen fluid was dispensed into centrifuge tubes and frozen.

### 2.3. Determination and screening of rumen fermentation parameters

Determination of the volatile fatty acid (VFA) concentration was carried out with reference to a previously published method (Erwin et al., 1961). The NH3-N concentration of rumen fluid was measured using the phenol sodium hypochlorite colorimetric method (Broderick and Kang, 1980). Ruminal fermentation parameters with strong covariance (variance inflation factor (VIF) > 10) were screened out by VIF analysis and subjected to redundancy analysis (RDA). The final rumen fermentation parameters that could be used for subsequent correlation analysis were obtained (Supplementary Table 2).

### 2.4. DNA Extraction, PCR amplification, and sequencing using Illumina Miseq

Total DNA from the microbial community in the ruminal fluid from 24 yaks was extracted according to the instructions of the FastDNA^®^ Spin Kit for Soil (MP Biomedicals, Solon, OH, United States). The determination of DNA concentration and purity, primer selection for PCR amplification, and PCR amplification procedures were performed according to our previous study (Yi et al., 2022). The PCR products of the same sample were mixed, subjected to 2% agarose gel to recover the PCR products, and extracted using an AxyPrep DNA Gel Extraction Kit (Axygen Biosciences, Union City, CA, USA). A Quantum Fluorometer (Promega, Madison, WI, USA) was used to detect and quantify the recovered products. The sequencing library was constructed and sequenced using the Illumina Miseq technique (Illumina, San Diego, CA, USA) in a manner consistent with our previous study (Yi et al., 2022). All raw sequences have been uploaded to the NCBI database with the accession number: PRJNA978645.

### 2.5. Sequencing data processing and analysis

Raw sequence quality control was carried out using fastp software (Chen et al., 2018), and splicing was performed using FLASH software (Magoc and Salzberg, 2011). The splicing process was carried out according our previously published methods (Yi et al., 2022). Based on the default parameters, after QC splicing, the optimized sequences were noise-reduced using the DADA2 (Callahan et al., 2016) plug-in in the Qiime2 process (Bolyen et al., 2019). Thereafter, the sequences were commonly referred to as amplicon sequence variants (ASVs). The number of sequences per sample after noise reduction was estimated according to the minimum value of the number of sample sequences, and the average coverage of each sample after leveling could reach 97.90%. Species taxonomy analysis of ASVs was performed based on the Silva 16S rRNA database (v 138) using the Naive bayes (or Vsearch, or Blast) classifier in Qiime2. The Majorbio cloud platform (a Qiime2 process) was used for the subsequent analysis.^1^

### 2.6. Metabolomics, data processing, and analysis

The extraction of rumen fluid metabolites, the analysis of extracted samples, and the analytical conditions for liquid chromatography and mass spectrometry (LC-MS) were the same as those in our previous study (Yi et al., 2022).

The LC-MS data were processed to obtain a data matrix (containing retention time, mass-to-charge ratio, and peak intensity). This data matrix was filled with vacant values using the 80% rule to remove missing values, i.e., variables with at least 80% of non-zero values were retained in at least one set of samples. Variables from quality control samples with a relative standard deviation (RSD) greater than 30% were removed and subjected to log_10_ logarithmic processing to obtain the final data matrix for subsequent analysis. The R package ropls (Version1.6.2) was used for principal component analysis (PCA). Partial least squares discriminant analysis (PLS-DA) and model stability were assessed using validation of the 7-cycle interaction. In addition, Student’s *t*-test analysis was performed. Student’s *t*-test *p*-values and the variable importance in the projection (VIP) values were used to select significantly differentially abundant metabolites, which were screened according to the criteria: VIP > 1 and *P* < 0.05. The above analyses were performed on the Majorbio Cloud Platform.*^seetextfootnote1^* Metabolic pathway enrichment analysis and topological analysis were performed on the identified significantly differentially abundant metabolites using MetabolAnalyst 5.0^2^ (Pang et al., 2022). The correlation network between rumen metabolites and the microbiota was visualized using the software Gephi 0.10.1.^3^

## 3. Results

### 3.1. Effect of supplemental feeding on growth performance and rumen fermentation parameters

Table 2 shows that final body weight (FBW) of the HCS group was higher than that of the LCS and CON groups. The average daily gain (ADG) in the four groups increased with the increase in supplementation. Table 2 demonstrates the effect of concentrate supplement feeding on rumen pH, NH_3_-N and VFAs. Rumen pH was lower in the MCS and HCS groups than in the CON and LCS groups (*P* < 0.01), while the NH_3_-N and total VFA concentrations were higher in the MCS and HCS groups than in the CON and LCS groups. Meanwhile, acetate concentrations were significantly lower in the MCS and HCS groups than in the CON and LCS groups. The propionate concentration was higher in the HCS group than in the other three groups, while the acetate:propionate ratio was lower in the HCS group than in the other three groups (*P* < 0.01). Valerate concentrations in the MCS and HCS groups were higher than those in the CON and LCS groups, while the valerate concentration in the HCS group were higher than that in the MCS group. Besides, isovalerate concentrations in the MCS and HCS groups were significantly higher than those in the CON group (*P* < 0.05).

**TABLE 2:** Concentrate supplementation affects performance and rumen fermentation parameters of cool-season grazing yaks.

Items^1^	Supplementary groups^2^				SEM	*P*-value
	CON	LCS	MCS	HCS		
**Production performance**						
IBW/kg	204.53	204.69	205.42	205.17	4.53	0.873
FBW/kg	187.37^c^	205.82^b^	246.21^a^^b^	278.42^a^	3.78	0.032
TWG/kg	−17.16^d^	1.13^c^	40.79^b^	73.25^a^	1.78	0.027
ADG/(g/d)	−235.17^d^	12.55^c^	453.32^b^	813.89^a^	33.31	0.003
**Rumen fermentation parameters**						
pH	6.87^a^	6.76^a^	6.46^b^	6.37^b^	0.049	<0.0001
NH_3_-N, mg/100 mL	7.65^c^	7.96^c^	11.04^b^	14.69^a^	0.699	<0.0001
Total VFA, mmol/L	49.25^b^	58.28^b^	64.90^a^	76.20^a^	3.078	0.0074
**VFA, %**						
Acetate	75.65^a^	74.05^a^	68.91^b^	66.33^b^	0.991	0.0001
Propionate	14.12^b^	13.69^b^	14.82^b^	18.26^a^	0.493	0.0005
Isobutyrate	0.83^b^	0.91^b^	1.45^a^	1.12^a^^b^	0.088	0.0406
Butyrate	8.06^b^	9.85^a^^b^	12.36^a^	11.84^a^	0.528	0.0062
Isovalerate	1.03^c^	1.15^bc^	1.81^a^	1.63^a^^b^	0.107	0.0146
Valerate	0.31^c^	0.33^c^	0.64^b^	0.83^a^	0.052	<0.0001
Acetate:Propionate	5.38^a^	5.43^a^	4.73^a^	3.71^b^	0.188	0.0003

^1^IBW, initial body weight; FBW, final body weight; TWG, total weight gain; ADG, average daily gain; VFA, volatile fatty acid. ^2^CON, control group; LCS, low concentrate supplement group; MCS, middle concentrate supplement group; HCS, high concentrate supplement group. ^a,b,c,d^The presence of the same letter after each treatment is non-significant, and the presence of completely different letters indicates a significant difference.

### 3.2. Ruminal bacterial diversity and composition

**FIGURE 1:**
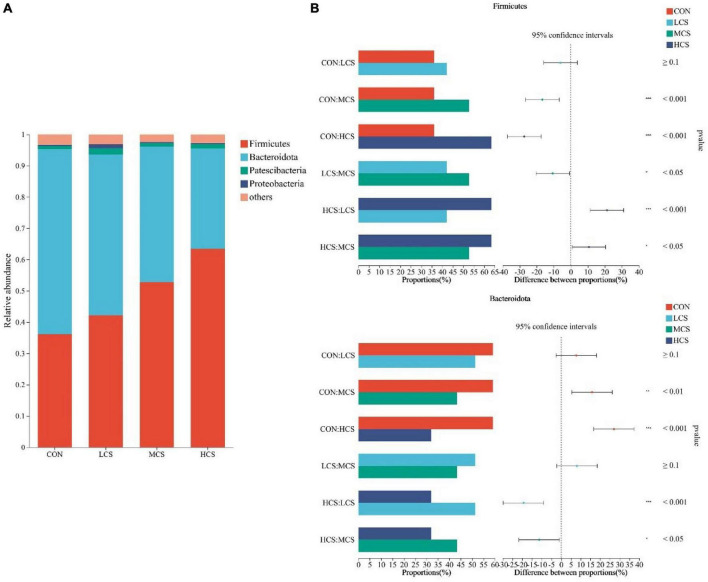
Composition of the rumen microbiota at bacterial phylum level **(A)** and differences between groups for major bacterial phyla **(B)**. CON, control group; LCS, low concentrate supplement group; MCS, middle concentrate supplement group; HCS, high concentrate supplement group. *0.01 ≤ *P* ≤ 0.05, **0.001 ≤ *P* ≤ 0.01, ***P ≤ 0.001.

**FIGURE 2:**
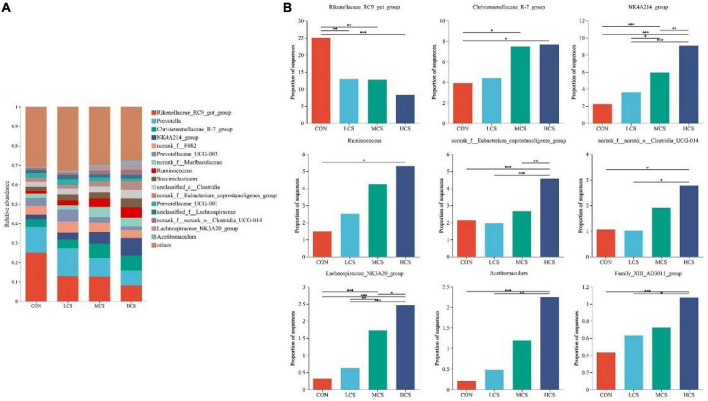
Composition of the rumen microbiota at bacterial genus level **(A)** and differences between groups for major bacterial genera **(B)**. *0.01 ≤ *P* ≤ 0.05, **0.001 ≤ *P* ≤ 0.01, ***P ≤ 0.001.

Ruminal fluid from 24 yaks was subjected to 16S rRNA high-throughput sequencing, which produced 1,430,293 optimized sequences, from which a total of 9,681 ASVs were obtained after noise reduction. After sampling the minimum number of sequences, 9,647 ASVs remained. Supplementary Figures 1A, B shows the bacterial communities’ α-diversity indices in the rumen of 24 yaks, including the Chao1 and Shannon indices. There was no significant difference in the α-diversity indices between the four groups. PCoA of the rumen bacterial communities showed that they were clearly differentiated between the four treatment groups (Supplementary Figure 1C). Figures 1, 2 depict the composition of the rumen at the bacterial phylum and genus levels, respectively. Twenty-one bacterial phyla and 281 bacterial genera were identified in the rumen bacterial flora, with *Firmicutes* (48.64%) and *Bacteroidota* (46.5%) as the dominant phyla (Figure 1A), and *Rikenellaceae_RC9_gut_group* (14.74%), *Prevotella* (11.17%), *Christensenellaceae_R-7_group* (5.88%), *NK4A214_group* (5.23%), *norank_f__F082* (4.77%), *Prevotellaceae_UCG-003* (3.75%), *norank_f__Muribaculaceae* (3.53%), and *Ruminococcus* (3.39%) as the dominant genera (Figure 2A).

### 3.3. Differences in ruminal bacterial species

Figures 1, 2 also demonstrate the differences in the relative abundance of bacterial communities in the rumen fluid samples among the four treatment groups. The abundance of *Firmicutes* in the rumen increased with the increasing supplementation level, while *Bacteroidota* showed the opposite trend. Among the four groups, the abundance of *Firmicutes* was highest in the HCS group and was higher in the MCS group than in the CON and LCS groups; meanwhile, the relative abundance of *Bacteroidota* in the HCS group was significantly lower than that in the other three groups, and that of *Bacteroidota* in the MCS group was significantly lower than that of the CON group (Figure 1B). Concentrate feeding significantly decreased the relative abundance of *Rikenellaceae_RC9_gut_group* at the genus level, while it increased the relative abundance of *Christensenellaceae_R-7_group*, *NK4A214_group*, *Ruminococcus*, *norank_f__ Eubacterium_coprostanoligenes_group*, *norank_f__norank_o__Clostridia_UCG-014*, *Lachnospiraceae_NK3A20_group*, *Acetitomaculum*, and *Family_ XIII_AD3011_group*. The abundance of *Rikenellaceae_RC9_gut_group* was lower than that of the CON group in all concentrate supplementation groups. The relative abundance of *norank_f__Eubacterium_coprostanoligenes_group* in the HCS group was significantly higher than that in the CON group, while the relative abundance of *norank_f__norank_o__Clostridia_UCG-014* in the HCS group was significantly higher than that in the other three groups. The abundance of *Acetitomaculum* and *Family_XIII_AD3011_group* in the HCS group was higher than that in the CON and LCS groups. Meanwhile, *Ruminococcus* abundance was higher in the HCS group than that in the CON group (Figure 2B).

### 3.4. Correlation of rumen differentially abundant genera with rumen fermentation parameters

**FIGURE 3:**
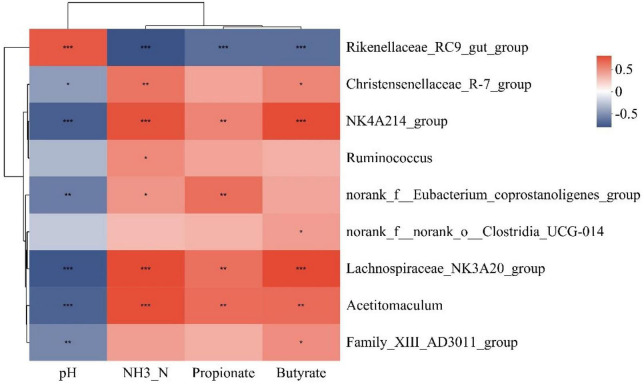
Heat map of correlation between rumen differential bacterial genus and screened rumen fermentation parameters. The color of the squares represents the magnitude of the correlation coefficients. *0.01 ≤ *P* ≤ 0.05, **0.001 ≤ *P* ≤ 0.01, ***P ≤ 0.001.

Supplementary Table 2 shows the significance of rumen fermentation parameters after VIF and RDA analysis. Among them, there were significant correlations (*P* < 0.05) between pH, NH_3_-N, propionate, and butyrate for subsequent analysis. Analysis of the species variability results showed that nine of the top 20 bacterial genera in the abundance ranking at the genus level of the rumen bacterial community differed between groups. Therefore, the above four rumen fermentation parameters were correlated with the nine differential bacterial genera based on Spearman’s correlation coefficient. The *Christensenellaceae_R-7_group* showed a positive correlation with NH_3_-N and butyrate levels, and a negative correlation with pH. The *norank_f__Eubacterium_coprostanoligenes_group* showed a significant positive correlation with both NH_3_-N and propionate levels, and a significant negative correlation with pH. In addition, NH3-N and propionate levels correlated positively with the abundances of *NK4A214_group*, *Acetitomaculum*, *Lachnospiraceae_NK3A20_group*, and *norank_f__Eubacterium_coprostanoligenes_group*, whereas pH correlated negatively correlated with the levels of these three bacterial genera. In contrast, *Rikenellaceae_RC9_gut_group* showed a significant negative correlation with NH_3_-N, propionate and butyrate levels, and a significant positive correlation with pH. In addition, *Ruminococcus* showed a positive correlation with NH_3_-N levels. Both *norank_f__norank_o__Clostridia_UCG-014* and *Family_XIII_AD3011_group* showed positive correlations with Butyrate levels, and the latter also showed a negative correlation with pH (Figure 3).

### 3.5. Comparative analysis of rumen metabolite samples

Supplementary Figure 2 depicts the intra-group similarity and inter-group variability of metabolites among the four groups of rumen fluid samples. PCA and PLS-DA analysis showed good intra-group aggregation and significant inter-group separation among the four groups of rumen fluid samples (Supplementary Figures 2A, B).

The correlation heat map of rumen fluid samples can indicate the degree of variability in metabolite composition and abundance between samples. Quantitative analysis can be performed by correlation analysis between samples, with correlations closer to 1 indicating a higher similarity in metabolic composition and abundance between samples (Supplementary Figure 2C). The heat map showed that the CON group showed a positive correlation with the LCS group and a significant negative correlation with the MCS and HCS groups. Meanwhile, the LCS group also showed a negative correlation with the MCS and HCS groups. With the increase of concentrate feeding, the degree of variation in metabolite composition and abundance in the MCS and HCS groups appeared to be significantly different from those in the CON and LCS groups, and there was a clear distinction.

### 3.6. Ruminal metabolite identification and differential metabolite analysis

Using LC-MS analysis of the four groups of rumen fluid samples, 1,453 metabolites were identified and screened by VIP > 1 and *P* < 0.05 (Supplementary Table 3). Table 3 lists the main metabolites that differed among the four treatment groups, and the metabolite concentrations of all six Metabolic classes in the table were significantly upregulated with increasing concentrate supplementation feeding (*p* < 0.05, fold-change (FC) > 1). The metabolite concentrations of all six metabolic classes in the table were significantly upregulated (*p* < 0.05, FC > 1) with the increase in concentrate supplementation feeding. In the HCS group, the concentrations of L-histidine, L-isoleucine, L-phenylalanine, L-tryptophan, L-tyrosine, L-valine, phosphocholine, deoxyadenosine monophosphate deoxyguanosine, deoxyinosine, xanthosine, guanosine, hypoxanthine, xanthine, histamine, uric acid, urocanic acid, choline, and phenylacetaldehyde were higher than in the other three groups. The concentrations of L-aspartic Acid, L-glutamate, L-histidine, L-isoleucine, L-phenylalanine, L-tryptophan, L-tyrosine, L-valine, N-acetyl-DL-glutamic acid, N-acetyl-L-ornithine, deoxyinosine, xanthosine, inosine, hypoxanthine, xanthine and urocanic acid were higher in MCS group than in CON and LCS groups. The concentrations of N-Acetyl-DL-Glutamic acid, Deoxyadenosine monophosphate, Deoxyguanosine, Guanine, Guanosine, Histamine, Uric acid, Choline Pantothenic Acid, and Phenylacetaldehyde were higher in the LCS group than in the CON group (Table 3).

**TABLE 3:** The main differentially abundant metabolites and their metabolic pathways among the four groups (VIP > 1, *p* < 0.01).

Metabolites	Fold Change^1^						Metabolic pathway
	CON vs. LCS	CON vs. MCS	CON vs. HCS	LCS vs. MCS	LCS vs. HCS	MCS vs. HCS	
**Amino acids, peptides, and analogs**							
L-Aspartic Acid	1.07	1.13	1.18	1.06	1.10	-	Aminoacyl-tRNA biosynthesis, Histidine metabolism, Arginine biosynthesis, Pantothenate and CoA biosynthesis.
L-Glutamate	1.05	1.12	1.15	1.07	1.10	-	Aminoacyl-tRNA biosynthesis, Histidine metabolism, Arginine biosynthesis.
L-Histidine	1.12	1.24	1.30	1.11	1.16	1.05	Aminoacyl-tRNA biosynthesis, Histidine metabolism.
L-Isoleucine	1.06	1.11	1.16	1.04	1.10	1.05	Aminoacyl-tRNA biosynthesis
L-Proline			1.09	1.03	1.07	1.04	
L-Tryptophan	1.08	1.15	1.24	1.07	1.15	1.08	
L-Phenylalanine	1.08	1.13	1.21	1.05	1.12	1.06	Aminoacyl-tRNA biosynthesis, Phenylalanine metabolism, Phenylalanine, tyrosine and tryptophan biosynthesis.
L-Tyrosine	1.07	1.13	1.20	1.06	1.13	1.07	
L-Valine	1.05	1.10	1.17	1.05	1.11	1.06	Aminoacyl-tRNA biosynthesis, Pantothenate and CoA biosynthesis.
N-Acetyl-DL-Glutamic acid	–	1.08	–	1.06	–	–	Arginine biosynthesis
N-Acetyl-L-ornithine	1.08	1.23	1.24	1.14	1.15	–	
**Lipids and lipid molecules**							
Phosphatidate [PA (10:0/i-18:0)]	1.14	1.34	1.39	–	–	–	Glycerophospholipid metabolism
Phosphatidylcholine	1.09	1.09	1.13	–	–	–	
Phosphocholine	1.20	1.27	1.40	–	1.16	1.10	
**Nucleosides, nucleotides and analogs**							
Adenosine	1.06	–	–	–	–		Purine metabolism
dAMP	–	–	1.19	1.09	1.21	1.10	
Deoxyguanosine	–	–	1.25	–	1.20	1.16	
Deoxyinosine	1.21	1.36	1.51	1.12	1.25	1.12	
Xanthosine	1.14	1.30	1.42	1.14	1.24	1.09	
Inosine	1.12	1.18	1.19	1.05	–	–	
Ribose 1-phosphate	1.08	1.12	–	–	–	–	
**Purine and purine derivatives**							
Guanine	–	–	1.08	–	1.07	–	Purine metabolism
Guanosine	–	–	1.13	–	1.09	1.06	
Hypoxanthine	1.07	1.12	1.14	1.04	1.06	1.02	
Xanthine	1.10	1.17	1.26	1.06	1.14	1.08	
**Organic nitrogen compounds**							
Histamine	–	–	1.16	–	1.13	1.10	Histidine metabolism
Uric acid	–	1.09	1.15	–	1.10	1.06	Purine metabolism. Histidine metabolism
Urocanic acid	1.09	1.21	1.28	1.11	1.17	1.05	Purine metabolism
**Others**							
Choline	–	1.05	1.08	–	1.05	1.03	Glycerophospholipid metabolism
Pantothenic acid	–	–	–	1.07	–	1.06	Pantothenate and CoA biosynthesis
Phenylacetaldehyde	–	–	1.09	–	1.08	1.05	Phenylalanine metabolism

^1^CON, control group; LCS, low concentrate supplement group; MCS, middle concentrate supplement group; HCS, high concentrate supplement group.

**FIGURE 4:**
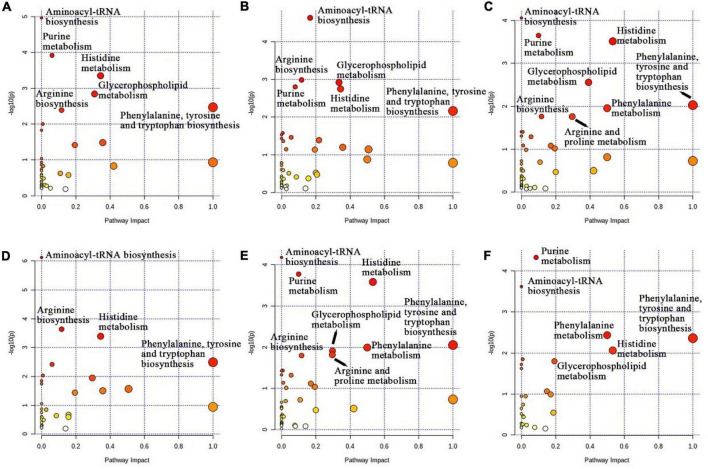
KEGG enrichment map of metabolic pathways. The *X*-axis indicates the pathway impact and the *Y*-axis indicates the degree of pathway enrichment. Larger circles represent higher pathway enrichment, and darker circles represent higher pathway impact values. **(A)** CON vs. LCM; **(B)** CON vs. MCS; **(C)** CON vs. HCS; **(D)** LCS vs. MCS; **(E)** LCS vs. HCS; **(F)** MCS vs. HCS. CON, control group; LCS, low concentrate supplement group; MCS, middle concentrate supplement group; HCS, high concentrate supplement group.

Topological analysis of metabolites showed that the main enrichment pathways of the four treatment groups were aminoacyl-tRNA biosynthesis; purine metabolism; histidine metabolism; arginine biosynthesis; phenylalanine, tyrosine and tryptophan biosynthesis; glycerophospholipid metabolism; phenylalanine metabolism; and arginine and proline metabolism (Figure 4). The enrichment pathways in the CON, LCS, and MCS groups were aminoacyl-tRNA biosynthesis, purine metabolism, histidine metabolism, arginine biosynthesis, glycerophospholipid metabolism and phenylalanine, tyrosine and tryptophan biosynthesis (Figures 4A, B). Similarly, the HCS group was enriched with the CON and LCS groups for phenylalanine, tyrosine and tryptophan biosynthesis; purine metabolism; histidine metabolism; glycerophospholipid metabolism; phenylalanine metabolism; arginine biosynthesis; arginine and proline metabolism; and aminoacyl-tRNA biosynthesis (Figures 4C, E). Figure 4D demonstrates the enrichment pathways of the LCS and MCS groups, including aminoacyl-tRNA biosynthesis, Arginine biosynthesis, histidine metabolism, and phenylalanine, tyrosine and tryptophan biosynthesis. The enrichment pathways of the LCS and MCS groups are shown in Figure 4F, including purine metabolism, aminoacyl-tRNA biosynthesis, phenylalanine metabolism, histidine metabolism, glycerophospholipid metabolism, and phenylalanine, tyrosine and tryptophan biosynthesis.

### 3.7. Correlation of rumen metabolites with rumen bacteria

**FIGURE 5:**
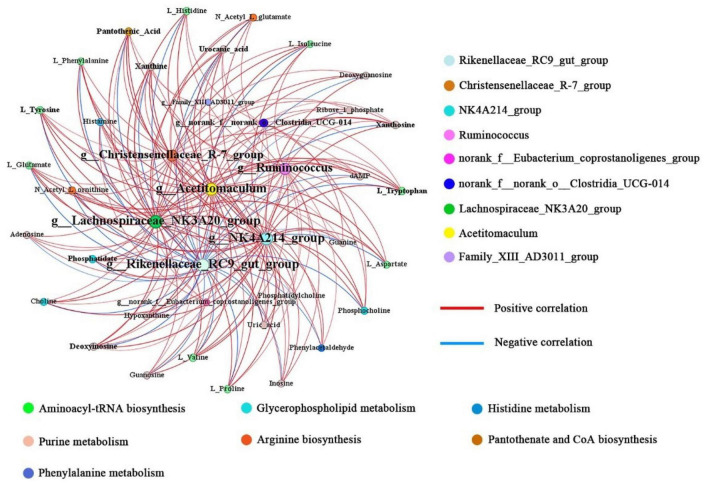
Networks associated with differentially abundant bacterial genera and differentially abundant metabolites. Node size represents the size of the degree; more edges connected, the larger the node.

To explore the potential relationships between rules bacterial communities and metals affected by concentrated feeding, we assessed correlations between nine affected rules bacterial general and major metals by Spearman correlation analysis, based on which we further mapped correlation networks (Supplementary Figure 3). Forty nodes and one hundred and eighty-nine edges formed a correlation network of rumen metabolites and microbial communities (Figure 5), of which 158 correlated positively and 31 correlated negatively (| r| >0.5, *P* < 0.05). The size of a node represents the degree of connectivity; the larger the node, the more nodes connected to it and the higher the degree of connection; by contrast, the fewer connected nodes, the lower the degree of connection. Among the nine bacterial genera, *Lachnospiraceae_NK3A20_group* and *Rikenellaceae_RC9_gut_group* were the most connected bacterial genera. The former belongs to the *Firmicutes* and had significant positive correlations with 31 metabolites, while the latter belongs to the *Bacteroidota* and also had significant negative correlations with 31 metabolites.

In addition, the four bacterial genera *NK4A214_group*, *Acetitomaculum*, *Ruminococcus*, and *Christensenellaceae_R-7_group* were also highly connected, with significant positive correlations with 30, 30, 26, and 25 metabolites, respectively. All four bacterial genera belong to the *Firmicutes*. By contrast, *norank_f__norank_o__Clostridia _UCG-014*, *Family_XIII_AD3011_group* and *norank_f__ Eubacterium_coprostanoligenes_group* had fewer positive correlations with metabolites, with significant positive correlations with 11, 1, and 4 metabolites, respectively. Therefore, based on the above results, we suggest that the abundance of *Firmicutes* in the rumen might have an important influence on the changes in rumen metabolite levels.

## 4. Discussion

In the cold season on the Tibetan plateau, the nutrient content of forage grasses is low and cannot provide yaks with the nutrients they need to grow properly (Sapkota et al., 2022). In this study, the CON group did not gain weight; however, the growth performance of the yaks improved significantly with higher concentrate supplement feeding, which is consistent with the results of a previous study (Long et al., 2005). This suggests that supplemental feeding can help yaks resist nutritional stress and improve their ability to adapt to their environment.

Ruminal fermentation parameters can reflect the reasons for the differences in growth performance of yaks. Among them, VFAs are fermentation products of microorganisms in the yak rumen, and their production depends on diet and the rumen microbiota (Sha et al., 2021). Total VFAs increased significantly and the pH decreased significantly with higher concentrate feeding. This is because of the high content of fermentable carbohydrates, such as starch, in the concentrate supplements. Their rapid degradation increases the concentration of total VFAs and lowers the pH. Insufficient availability of forage in the cold season and low quality of forage can lead to lower dry matter intake of grazing yaks (Sapkota et al., 2022), which might be responsible for the lower concentration of total VFAs in the CON group relative to the other groups. Previous studies found that an increase in concentrate supplement feeding increased the concentration of VFAs other than acetate, but decreased the concentration of acetate and the ratio of acetate to propionate (Liu et al., 2019; Yi et al., 2022). The acetate:propionate ratio reflects the efficiency of feed energy use by animals, and a decrease in the ratio represents an increase in the efficiency of feed energy use by yaks (Chen et al., 2021), which is consistent with the significant increase in growth performance of yaks fed concentrate supplements. This change in the acetate:propionate ratio could provide guidance for future cold-season grazing yak production. In addition, isobutyrate and isovalerate in the rumen originate from feed protein degradation and amino acid deamination (Eugène et al., 2004), and their increase reflected the increased protein content in the rumen of the yaks after supplementation, which also led to an increase in the ruminal NH_3_-N content. Meanwhile, elevation of NH_3_-N, the main source of nitrogen for rumen microbial protein synthesis, was accompanied by an increase in microbial protein production in the rumen (Zhang et al., 2017a). Therefore, we concluded that on the one hand, feeding of concentrate supplements can alter the available fermentation substrates in the yak rumen, resulting in significant fluctuations in rumen fermentation parameters. On the other hand, changes in fermentation substrates can affect ruminal homeostasis, thereby affecting rumen microorganisms and their metabolic pathways (Ghimire et al., 2017). Therefore, changes in rumen pH, VFAs, and NH3-N might be related to changes in the rumen bacterial community of yaks.

In the present study, the most dominant bacterial phyla in the rumen of grazing yaks were *Firmicutes* and *Bacteroides*, accounting for approximately 95% of all bacterial phyla, which agreed with the results of previous studies (Liu et al., 2019; Yi et al., 2022). The abundance of *Firmicutes* increased with increasing concentrate supplementation, while the opposite was true for *Bacteroidota*. This change in *Firmicutes* can be explained by the fact that concentrate supplementation added a large amount of energy, and *Firmicutes* have the effect of absorbing a large amount of energy from a small amount of food (Liao et al., 2023). The decrease in abundance of *Bacteroidota* was attributed to the decrease in structural polysaccharides in the rumen caused by the increase in concentrate supplementation. Corroboratively, *Bacteroidota* are the main degraders of structural polysaccharides in the rumen (Lu et al., 2023).

At the genus level, we detected nine bacterial genera with significant differences (proportion of sequences > 1%). The *Rikenellaceae_RC9_gut_group* is a member of the *Rikenellaceae* family, which is involved in the degradation of fibrous polysaccharides (Zhang et al., 2022b,^2023^). Feeding of concentrate supplements led to a decrease in the concentration of fibrous polysaccharides in the rumen, which could also explain the correlation between *Rikenellaceae_RC9_gut_group* and rumen fermentation parameters. The results of a study on the differences in rumen microbial composition between house-fed yaks (high protein diet) and grazing yaks showed that *Christensenellaceae_R-7_group* was significantly more abundant in the rumen of the house-fed yaks than in in the grazing yaks (Yao et al., 2022), which is consistent with the results of the present study. *Christensenellaceae_R-7_group*, as a probiotic in the rumen, promotes host digestion and their absorption of nutrients or improves feed utilization (Ghimire et al., 2017), which was confirmed by its positive correlation with NH_3_-N and butyrate. The relative abundance of *NK4A214_group*, *Acetitomaculum*, and *Lachnospiraceae_NK3A20_group* all increased with increasing addition of concentrate supplementation, which is generally consistent with the results of previous studies (Liu et al., 2019; Sha et al., 2021; Yi et al., 2022). Among them, the *NK4A214_group* belongs to the *Ruminococcaceae* family, which plays an important role in the digestion of resistant starch (Anderson et al., 2016; Ferrario et al., 2017), and the feeding of concentrate supplements would deliver more resistant starch to the rumen. This might also be the main reason for the positive correlation between the *NK4A214_ group* and the concentrations of propionate and butyrate. Like the *NK4A214_ group*, *Ruminococcus* belongs to the *Ruminococcaceae* family. The abundance of *Ruminococcus* was highest in the HCS group, correlated positively with NH_3_-N levels, which is consistent with a previous report (Ahmad et al., 2021). The reason for this phenomenon might be that the difference in abundance of *Ruminococcus* was not significant among the groups other than in the HCS group. *Lachnospiraceae_NK3A20_group* and *Acetitomaculum* both belong to the *Lachnospiraceae* family, which is involved in the metabolism of many carbohydrates (e.g., starch) in the rumen (Ravelo et al., 2022, 2023). This is the reason why the abundance of *Lachnospiraceae_NK3A20_group* and *Acetitomaculum* increased significantly with the increase of concentrate supplement feeding. In addition, they can produce butyrate and other short-chain fatty acids by hydrolyzing carbohydrates such as starch (Reichardt et al., 2014; Chen et al., 2017), which is also consistent with the result that *Lachnospiraceae_NK3A20_group* and *Acetitomaculum* were significantly and positively correlated with propionate and butyrate. The *norank_f__Eubacterium_coprostanoligenes_group* can breakdown cholesterol into fecal sterols, thus playing an important role in lowering intestinal cholesterol levels in animals (Macdonald et al., 1983). The abundance of *norank_f__Eubacterium_coprostanoligenes_group* was higher in the HCS group than in the CON and LCS groups, presumably because the increased concentrate supplementation introduced a large amount of cholesterol into the rumen of the yaks in the HCS group. Both *norank_f__norank_o__Clostridia_UCG-014* and *Family_XIII_AD3011_group* belong to *Clostridia* Class in the *Firmicutes* phylum, and their changes in abundance tended to be consistent with those of the *Firmicutes* phylum. Meanwhile, their significant positive correlation with butyrate suggested that they might be butyrate-producing bacteria.

In this study, we used metabolomics to fully reveal the effects of supplemental feeding on rumen metabolites in grazing yaks. Our previous study found that changes in diet structure altered the rumen environment and had a greater impact on the rumen microbial community (Yi et al., 2022). In the present study, based on the results of rumen fermentation parameters and microbial diversity, we suggest that changes in rumen metabolites might occur. The amino acids in the rumen are mainly derived from the degradation of feed proteins and microproteins by resident microorganisms. We observed that L-phenylalanine and L-tyrosine levels were significantly upregulated with increased concentrate supplement feeding. These two amino acids could be used as markers of protein metabolism (Liu et al., 2019; Xu et al., 2021), suggesting that feeding concentrate supplements brought sufficient amino acid reserves to yaks and enhanced their protein synthesis. This represents a new insight into yak production, in which supplemental feeding in the cool season mobilizes protein synthesis and metabolism in grazing yaks, which might promote muscle growth. In addition, L-valine and L-glutamic acid were also significantly upregulated with higher supplementation concentrations, and the former can be metabolized in the rumen to produce isobutyrate and isovalerate, which act as potential ketogenic and glucogenic substances (Menahan and Schultz, 1964). This also explains the significant increase in isobutyrate and isovalerate concentrations in previous studies of rumen fermentation parameters (Liu et al., 2019; Yi et al., 2022). Meanwhile, L-isoleucine, which is a branched chain amino acid (BCAA), together with L-valine, was also significantly upregulated in the supplemented group. BCAAs can repair muscles, control blood glucose, and provide energy to body tissues (Buse and Weigand, 1976; Freund and Fischer, 1978), indicating that supplementation could help repair muscle damage caused by antinutritional stress to some extent. L-histidine has been reported to be the third limiting amino acid in ruminants after L-lysine and L-methionine (Van den Bossche et al., 2023), and its increased content helps to regulate ruminant nitrogen utilization and improve counterproductive performance (Morris and Kononoff, 2020). L-proline is a functional amino acid (Wu et al., 2011) whose levels in the yak rumen have been reported to increase with increasing levels of dietary concentrates (Zhang et al., 2017b), which is in agreement with the results of the present study. The concentration of L-aspartate increased significantly in the supplemented groups, probably because the supplementation provided a large source of nitrogen to the yaks. L-aspartic acid participates in the ornithine cycle and promotes the production of urea from oxygen and carbon dioxide, thereby reducing the amount of nitrogen and carbon dioxide in the blood (Peng et al., 2019). In addition, L-aspartic acid, N-acetyl-DL-glutamic acid and N-acetyl-L-ornithine are all involved in the biosynthesis of L-arginine, which is also an important player in the urea cycle (Lerzynski et al., 2006). L-tryptophan can regulate the immune function of ruminant organism (Patil et al., 2013), and its concentration was significantly upregulated in the supplemented group, indicating that supplementation contributes to improving the immunity of the organism. All of the above amino acids are involved in the aminoacyl-tRNA biosynthesis process, which is a step that all amino acids must undergo before they can be incorporated into the peptide chain. Amino acids undergo an activation reaction catalyzed by the synthesis of aminoacyl-tRNA enzymes for energy, and the amino acids then bind to tRNAs to form aminoacyl-tRNA, which provides the material basis for protein synthesis (Li et al., 1997). The enrichment of this process once again proves that feeding concentrate supplements enhances protein synthesis and metabolism in yaks.

Glycerophospholipids participate in protein recognition and signal transduction in animal cell membranes (Hayashi and Dennis, 2023). Supplemental feeding of concentrate increased the levels of metabolites associated with glycerophospholipid metabolism (phosphatidate, phosphatidylcholine, phosphocholine, and choline) in the yak rumen, which might be associated with enhanced protein metabolism in yaks organism. The concentration of pantothenic acid was upregulated in the concentrate supplemented, probably because the concentrate supplement enhanced fatty acid and pyruvate metabolism in yaks (Tahiliani and Beinlich, 1991; Abo Alrob and Lopaschuk, 2014). Biogenic amines (e.g., spermine, tyramine, and histamine) regulate the synthesis of DNA, RNA, and proteins, as well as maintain the stability of biological membranes (Wojcik et al., 2021). In the present study, histamine levels were significantly upregulated in the HCS group compared with those in the CON group, which is consistent with the results of a previous study (Humer et al., 2018).

Previous studies have found that increased biogenic amine production in ruminants is closely associated with an acidic rumen environment (Zhang et al., 2022a), suggesting that our supplementation of concentrates might lead to an overly acidic ruminal environment in yaks, reminding us to take steps to ensure animal health during production.

Notably, a number of metabolites associated with purine metabolism showed changes in their concentrations. Nucleic acids are synthesized by microbial populations in the rumen by degrading nitrogen from the feed (Fujihara and Shem, 2011). These metabolites were found in higher levels in three concentrate supplementation groups in the present study. Xanthine and hypoxanthine, products of nucleic acid degradation by gram-negative bacteria (Zhang et al., 2022a), were elevated in the high concentrate diet. Additionally, purines are converted into hypoxanthine by intermediates, and hypoxanthine is transformed into xanthine by secondary metabolism. Thus, xanthine and hypoxanthine are capable of acting as biomarkers of microbial protein synthesis (Ametaj et al., 2010; Zhang et al., 2017b). According to these findings, concentrate supplements are beneficial for purine metabolism in cool-season grazing yaks. Moreover, both *Lachnospiraceae_NK3A20_group* and *Acetitomaculum* are gram-negative bacteria that correlate positively with purine metabolites. Their relative abundance was upregulated in the concentrate supplement groups. This suggested that the feeding with the concentrate supplement accelerated the microbial passaging process in the rumen, which might be responsible for the increase in the number of bacterial degradation products. The *NK4A214_ group*, *Acetitomaculum*, and *Lachnospiraceae_NK3A20_ group* all correlated positively with the levels of differentially abundant metabolites. Similarly, *Christensenellaceae_R-7_group* and *Ruminococcus* also showed a positive correlation with the levels of most differentially abundant metabolites. This indicated that these five bacterial genera are more active in the degradation of the concentrate supplements. In addition, *Rikenellaceae_RC9_gut_group* showed a negative correlation with the levels of all differentially abundant metabolites, which was associated with a decrease in their relative abundance with increasing supplementation levels.

## 5. Conclusion

This study used multi-omics techniques to explore the dynamics of rumen bacterial communities and metabolites in cool season grazing yaks that received supplemental feeding. Changes in rumen bacterial communities and metabolites, and correlations among them, might affect the productivity of cool-season grazing yaks. Our comprehensive analysis revealed the association between the rumen environment and bacterial genera. These findings showed that diet influences the function of the rumen microbial community in a complex manner, and suggest that high-dose supplementation of yaks during the cold season can avoid nutritional stress and achieve good economic returns. This study provides a theoretical basis for developing an appropriate cold season supplemental feeding program for yaks.

## Data availability statement

The datasets presented in this study can be found in online repositories. The names of the repository/repositories and accession number(s) can be found in this article/Supplementary material.

## Ethics statement

The animal study was approved by the Experimental Animal Welfare and Animal Experimentation Ethics Committee of China Agricultural University. The study was conducted in accordance with the local legislation and institutional requirements.

## Author contributions

HW, SL, SC, and ZZ designed the study. SY and DD performed the sample process. SY performed the data analysis and wrote the manuscript. HW and ZZ revised the manuscript. All authors have read and approved the final manuscript.

## Footnotes

1
https://www.majorbio.com

2
http://www.metaboanalyst.ca

3
https://gephi.org/

## Supplementary material

Supplementary Figure 1 Alpha diversity and beta diversity values for the four treatment groups.

Supplementary Figure 2 PCA plots, PLS-DA plots, and sample correlation heat maps for the four treatment groups.

Supplementary Figure 3 Spearman’s correlation coefficient between differential bacterial genera and rumen metabolites.

Supplementary Table 1 Nutritional value of forage grasses during the experiment.

Supplementary Table 2 Ruminal fermentation parameters after variance inflation factor (VIF) analysis and RDA analysis.

Supplementary Table 3 List of identified metabolites.
