# Supplementary figures and images for: Concentrate supplementation improves cold-season environmental fitness of grazing yaks: responsive changes in the rumen microbiota and metabolome
Source: Front Microbiol. 2023 Aug 28;14:1247251. doi: 10.3389/fmicb.2023.1247251 (PMC10494446; doi:10.3389/fmicb.2023.1247251)

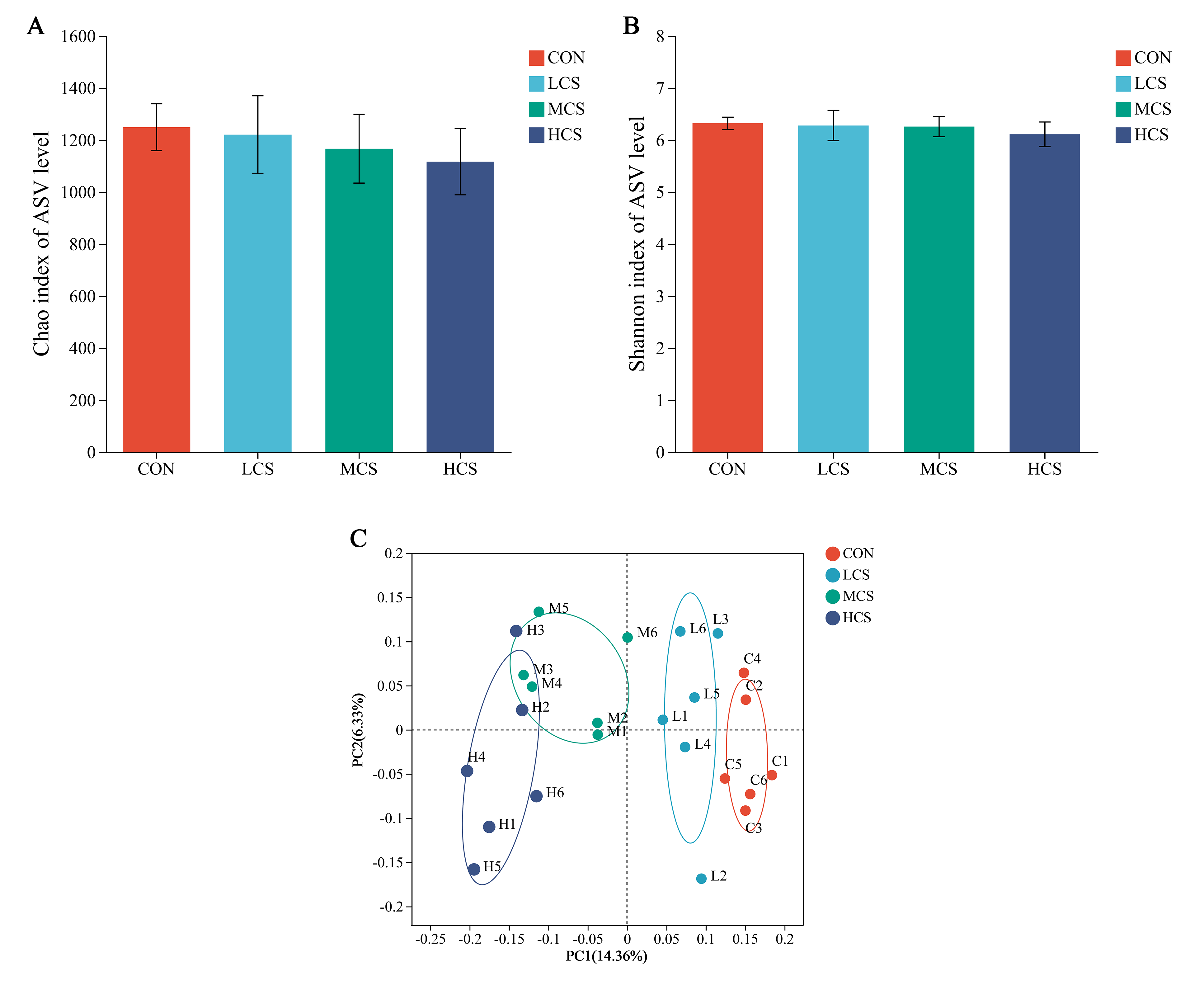

Supplement: Supplementary Figure 1 — Alpha diversity and beta diversity values for the four treatment groups. [file Image_1.PNG]

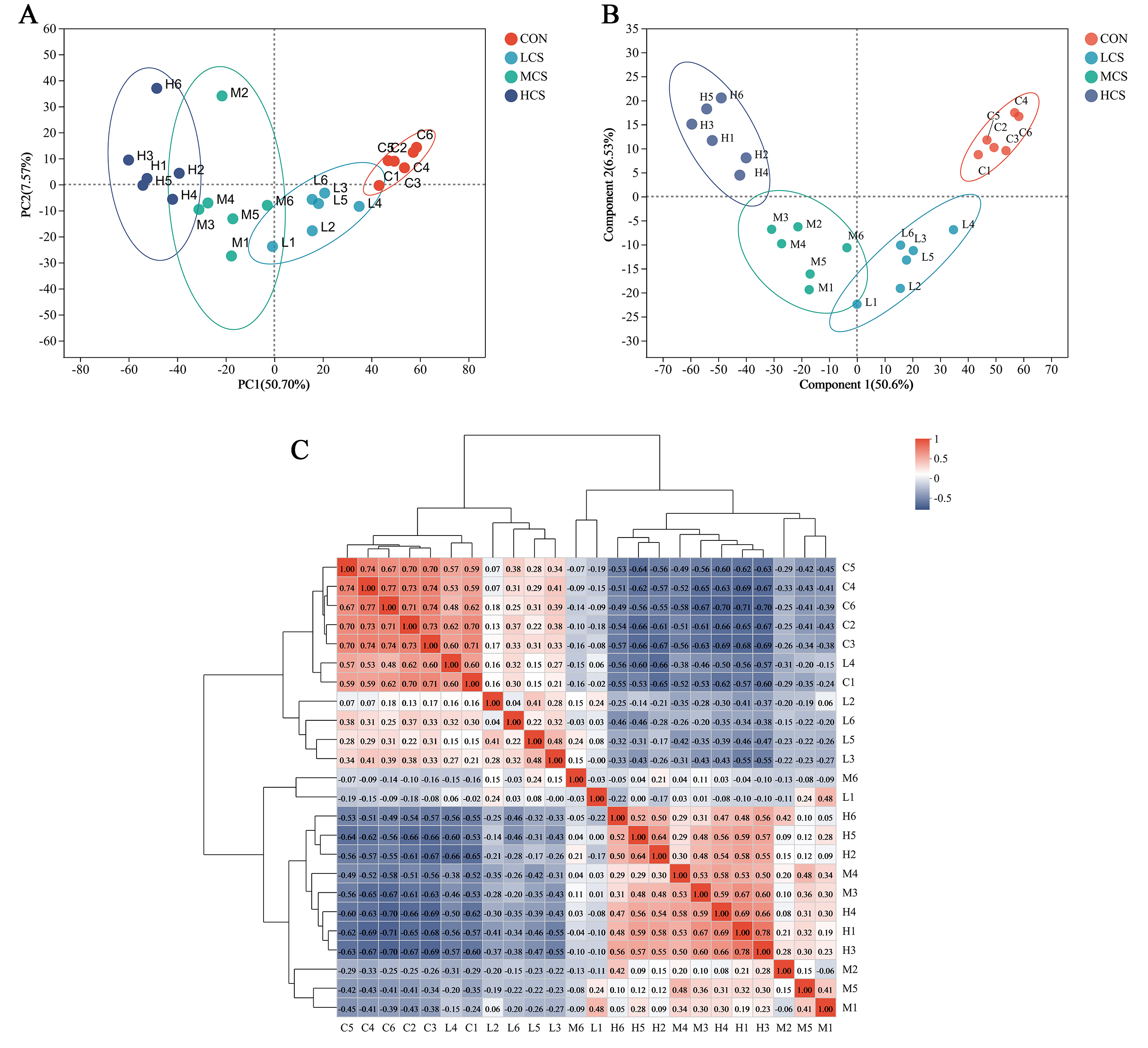

Supplement: Supplementary Figure 2 — PCA plots, PLS-DA plots, and sample correlation heat maps for the four treatment groups. [file Image_2.PNG]

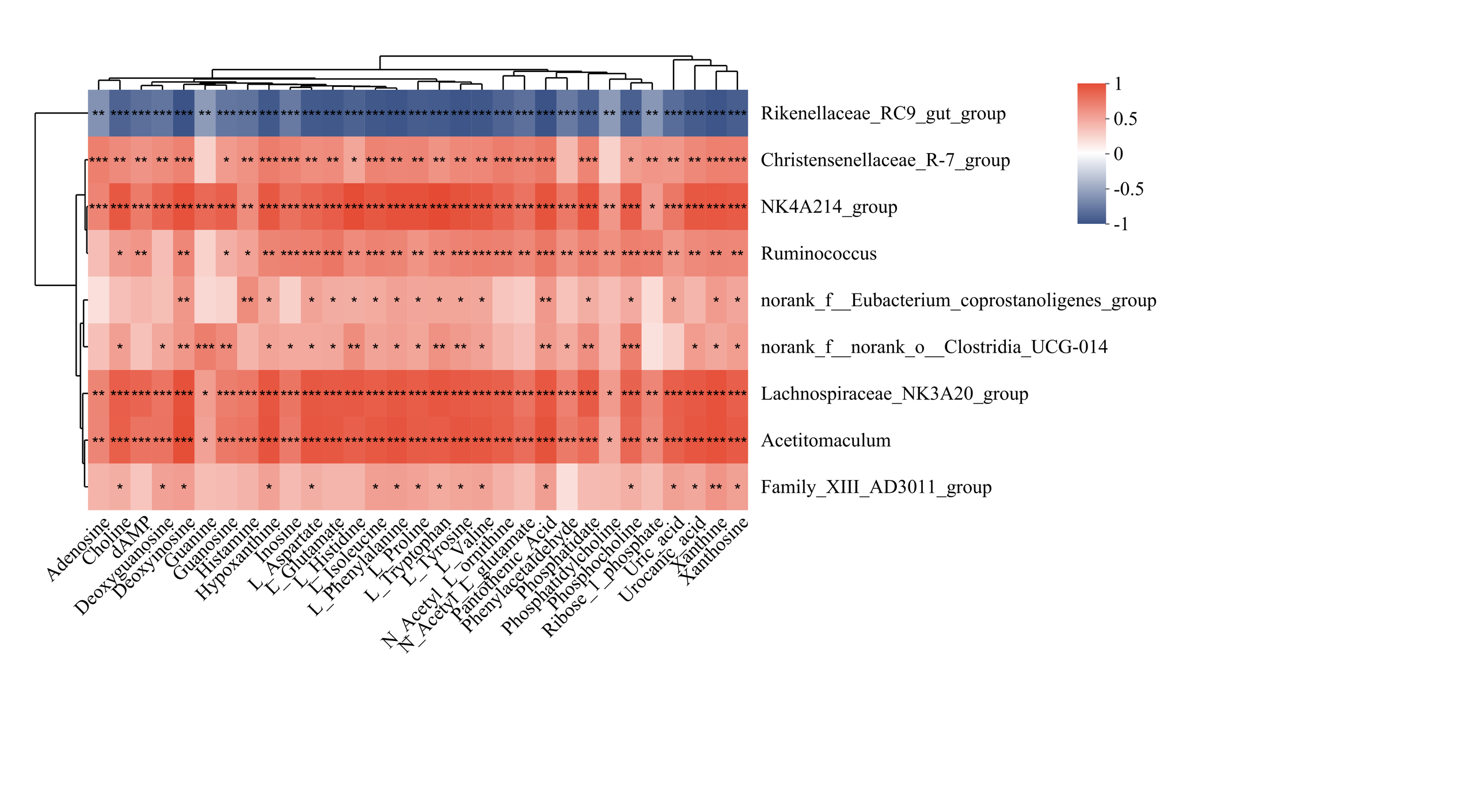

Supplement: Supplementary Figure 3 — Spearman’s correlation coefficient between differential bacterial genera and rumen metabolites. [file Image_3.PNG]
