# Supplementary material for: Concentrate supplementation improves cold-season environmental fitness of grazing yaks: responsive changes in the rumen microbiota and metabolome
Source: Front Microbiol. 2023 Aug 28;14:1247251. doi: 10.3389/fmicb.2023.1247251 (PMC10494446; doi:10.3389/fmicb.2023.1247251)
Supplement: Supplementary Table 1 — Nutritional value of forage grasses during the experiment. [file Table_1.docx]

**Supplementary Table** Chemical composition of pasture (DM basis, %)

| Chemical composition^1^ | Content |
| --- | --- |
| DM | 96.52 |
| CP | 8.91 |
| NDF | 64.23 |
| ADF | 33.55 |
| Crude fat | 2.37 |
| Ca | 0.50 |
| P | 0.06 |

^1^ DM, dry matter; CP, crude protein; NDF, neutral detergent fiber; ADF, acid detergent fiber
