# Supplementary material for: Concentrate supplementation improves cold-season environmental fitness of grazing yaks: responsive changes in the rumen microbiota and metabolome
Source: Front Microbiol. 2023 Aug 28;14:1247251. doi: 10.3389/fmicb.2023.1247251 (PMC10494446; doi:10.3389/fmicb.2023.1247251)
Supplement: Supplementary Table 2 — Ruminal fermentation parameters after variance inflation factor (VIF) analysis and RDA analysis. [file Table_2.docx]

**Supplementary Table 4** VIF variance inflation factor analysis and RDA/CCA analysis of rumen fermentation parameters

| Items^1^ | pH | NH_3_-N | Acetate | Propionate | Isobutyrate | Butyrate | Isovalerate | Valerate |
| --- | --- | --- | --- | --- | --- | --- | --- | --- |
| **VIF variance inflation factor analysis** | | | | | | | | |
| BSVIF | 4.442 | 2.782 | 5473.571 | 1510.333 | 64.085 | 1709.054 | 76.519 | NA |
| ASVIF | 4.407 | 2.121 | - | 2.610 | 2.233 | 4.160 | - | - |
| **RDA/CCA analysis** | | | | | | | | |
| RDA1 | 1 | -0.9981 | - | -0.9837 | -0.9546 | -0.981 | - | - |
| RDA2 | 0.0079 | 0.0618 | - | -0.1796 | 0.298 | 0.194 | - | - |
| r^2^ | 0.4627 | 0.6062 | - | 0.3742 | 0.1482 | 0.2662 | - | - |
| *p*-value | 0.002 | 0.001 | - | 0.016 | 0.183 | 0.047 | - | - |

^1^BSVIF, VIF values of rumen fermentation parameters before screening; ASVIF, VIF values of rumen fermentation parameters after screening; RDA,

RDA列表示环境因子与排序轴的相关性大小；r2值表示环境因子与物种分布的决定系数（大小范围0-1），该值越小则该环境因子对物种分布的影响越小；p_values代表相关性的显著性检验值，p_values<0.05，表示具有显著相关性
